# Supplementary material for: Netcombin: An algorithm for constructing optimal phylogenetic network from rooted triplets
Source: PLoS One. 2020 Sep 18;15(9):e0227842. doi: 10.1371/journal.pone.0227842 (PMC7500971; doi:10.1371/journal.pone.0227842)
Supplement: S4 File — (ZIP) [file pone.0227842.s004.zip › SequenceData-Triplet/sequenceData-10-20-30-0-Triplet/length 200, numseq 30, rate 10^-7/1 seq.pdf]

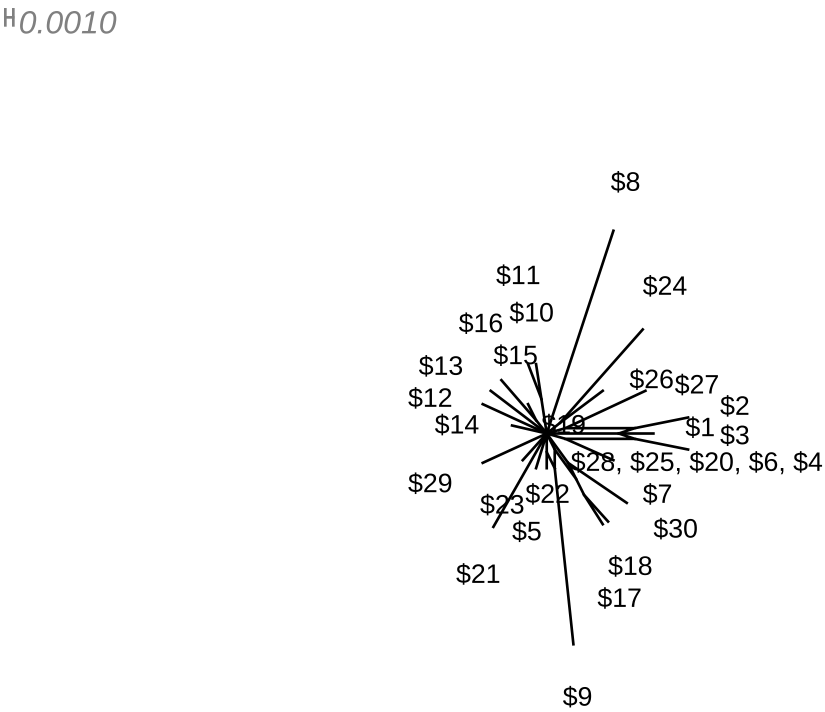

H 0.0010
$8
$11
$24
$10
$16
$15
$13
$26
$27
$12
$2
$19
$14
$1
$3
$28, $25, $20, $6, $4
$29
$7
$22
$23
$5
$30
$21
$18
$17
$9
